# Mechanistic insights into the antiproliferative effect of the redox-active iron chelator Dp44mT on multiple myeloma cell lines

**DOI:** 10.1016/j.htct.2025.106233

**Published:** 2025-12-23

**Authors:** Aarti Sharma, Latha Pathangey, Sinto Sebastian Chirackal, Kiran K. Mangalaparthi, Akhilesh Pandey, Rafael Fonseca, Sundararaman Swaminathan

**Affiliations:** a Mayo Clinic Arizona USA; b Department of Nephrology, Indian Institute of Science, Bengaluru, Karnataka, India; c Division of Hematology and Medical Oncology, Mayo Clinic Phoenix, AZ, USA; d Department of Laboratory Medicine and Pathology, Mayo Clinic, Rochester, MN, USA; e Mayo Clinic Comprehensive Cancer Center, Mayo Clinic, Scottsdale, AZ, USA; f Manipal Academy of Higher Education, Manipal, Karnataka, India; g Center for Individualized Medicine, Mayo Clinic, Rochester, MN, USA

**Keywords:** Dp44mT, Iron chelators, Proteomics, Phosphoproteomics, Reactive oxygen species, Oxidative stress, Multiple myeloma

## Abstract

**Background:**

Impaired iron metabolism has been linked to the pathogenesis of multiple myeloma. Redox active iron chelators have gained attention as potential anti-cancer agents as they target the high iron dependency of cancer cells. This study explored the potential mechanisms underlying the anti-multiple myeloma effect of the redox active iron chelator Dp44mT (Di-2-pyridylketone 4,4-dimethyl-3-thiosemicarbazone).

**Methods:**

The effect of Dp44mT was tested on both immunomodulatory drug-sensitive and drug-resistant multiple myeloma cell lines using the MTT assay. Proteomic and phosphoproteomics characterization were utilized to explore the mechanisms of Dp44mT action on multiple myeloma cells. In addition, a real-time polymerase chain reaction assay was performed to examine the expressions of major iron metabolism genes. Reactive oxygen species, lipid peroxidation, mitochondrial membrane potential, and intracellular iron compartmentalization were measured using flow-cytometry.

**Results:**

The high potency of Dp44mT in killing multiple myeloma cell lines was confirmed. Treatment with Dp44mT showed evidence of deregulated cellular iron metabolism, reactive oxygen species homeostasis, and mitochondrial membrane potential in multiple myeloma cell lines. As possible mechanistic pathways of Dp44mT, there was overrepresentation of the AMPK pathway, cell cycle, endoplasmic stress, and down regulation of *ACSL4* (*acyl-CoA synthetase long chain family member 4*).

**Conclusion:**

This study suggests an in vitro, anti-multiple myeloma effect of Dp44mT that appears to be mediated by dysregulated iron metabolism, reactive oxygen species, and other biological pathways.

## Introduction

Multiple myeloma (MM) is a plasma cell malignancy characterized by excessive production of circulating monoclonal immunoglobulin. Acquired resistance to the available treatment regimens poses the largest problem in disease management. Identifying the mechanisms and developing strategies to overcome resistance is key for improving disease outcomes.

Iron plays a crucial role in various cellular processes, including DNA synthesis, oxygen transport, and energy production under a tight regulatory system. The pathogenesis of MM has consistently been linked with deregulated iron metabolism [1]. High availability of intracellular iron can augment the growth of cancer cells and boost the dissemination cascade. However, under certain conditions, iron also promotes the production of toxic reactive oxygen species (ROS) that initiate ferroptosis (a form of cell death mediated by iron-induced lipid peroxidation) as a defense against cancer [2]. Thus, iron metabolism is tightly regulated to avoid the detrimental effects of iron induced toxicity while preserving its vital role in healthy cells.

Recently, a class of redox-active iron chelators that bind and eliminate excess iron from the cells, and yet have the potential to generate ROS to kill cancer cells has been investigated as possible therapeutic agents in hematological malignancies. Multiple redox-active iron chelators, like di-2-pyridyl thiosemicarbazone analogues, have been studied for their favorable applications in cancer treatment. Among them, Dp44mT (di-2-pyridylketone-4,4,-dimethyl-3-thiosemicarbazone), an iron chelating complex, has shown promise as an anti-tumor agent in both in-vitro and in-vivo models [[3], [4], [5]]. Previous studies have also established Dp44mT as a potential inducer of apoptosis as a single agent in many different cancers including, breast, osteosarcoma, neuroepithelioma, melanoma, glioblastoma and oral squamous cell carcinoma [4,[6], [7], [8], [9]]. A preliminary study investigated the effects of Dp44mT in MM cell lines, finding that Dp44mT inhibited the growth and viability of MM cells, induced cell cycle arrest, and promoted apoptosis [3]. Although Dp44mT has shown promising results in preclinical models, further research including human clinical trial studies are required to establish its suitability in the clinical setting.

Despite its potential as an anti-cancer agent, the precise molecular targets and pathways through which Dp44mT exerts its effects are underexplored. This study undertakes a comprehensive evaluation of the anti-cancer effects of potent iron chelators in MM. A multi-modal experimental approach was employed including high-resolution mass spectrometry for proteomics and phosphoproteomics profiling, cell viability assays to assess cytotoxicity and proliferation, and a range of flow cytometry approaches to monitor ROS, lipid peroxidation, mitochondrial membrane potential (ΔΨm), and intracellular iron compartmentalization. Taken together, the potent anti-cancer effect of Dp44mT in MM and the intracellular mechanism of its anti-MM effect were established by the utilization of advanced mass spectrometry-based technologies.

## Methods

### Study design

The experimental plan of this study involved a comprehensive investigation of a wide range of anti-cancer compounds in eight different MM cell lines. MM cells showed high sensitivity to iron chelating drugs independent of immunomodulatory drug (IMiD) sensitivity as IMiD resistant cells were also susceptible to treatment. To understand the detailed mechanism involved behind anti-MM activity of redox-active iron chelating drugs, two cell lines one based on IMiD sensitivity (*MM.1S*) and another on resistance (*RPMI-8226*) were selected for further study. Dp44mT was most effective in inducing MM cell death and was therefore chosen for further studies. To identify mechanisms, a highly advanced approach was used to establish the total proteomics and phosphoproteomics signatures influenced by Dp44mT treatment.

### Cell culture

The human MM cell lines *JJN3, RPMI-8226, MM.1S* and *KMS11* were cultured in Roswell Park Memorial Institute (RPMI) 1640 medium containing 10 % AB serum (Gem cell human) and 1 % penicillin‒90 streptomycin (P/S) solution (Gibco) at 37 °C in a humidified incubator (SANYO, Japan) containing 5 % CO_2_.

### RNA extraction, cDNA synthesis and real-time polymerase chain reaction

Total RNA was extracted using Qiagen total RNA extraction kit following the manufacturer’s recommended protocol. cDNA was synthesized by utilizing iscript cDNA synthesis kit (BioRad). Real-time polymerase chain reaction (RTqPCR) assay was performed using Fast SYBR Green Master mix (Applied Biosystems) in 20 µL total volume. The cDNA was diluted 10 fold to carry out RTqPCR amplification. The final concentration of each primer in the reaction mix was 0.5 µM. Reactions were run in a RTqPCR System (LightCycler® 480 Instrument II Roche). The cycling conditions were set at 95 °C for 25 s, 35 cycles at 95 °C for 1 s, 60 °C for 20 s, 95 °C for 15 s, 60 °C for 1 minute, and a gradient from 60 °C to 95 °C with a continuous detection at 0.015 °C/s increases for 15 min.

### Cell proliferation assay

The cell proliferation assay was carried out using the MTT 3-(4,5-dimethylthiazol-2-yl)-2,5-diphenyltetrazolium bromide) reagent. The drugs Lenalidomide (Cat#SML2283), Salinomycin, Di-2-pyridylketone (DpC, Cat#SML0483) and Di-2-Pyridylketone 4,4-Dimethyl-3-Thiosemicarbazone, (Dp44mT, Cat#SML0186), were obtained from sigma Aldrich Burlington, MA, United States in lyophilized form and were reconstituted at the desired concentrations as instructed by manufacturer: small single use aliquots were stored at −80 °C until further use. The different MM cells were independently seeded into 96-well plates at a density of 3 × 10^4^ cells per well. After seeding, cells were treated with various drugs according to the planned experimental workflow and cultured for up to 72 h. After 72 h, 15 µL of the MTT reagent were added per well followed by incubation for 4 h at 37 °C in a humidified incubator. One hundred microliters of stop solution were added to each well to dissolve the formazan crystals. The microplate was then incubated at 37 °C to dissolve the formazan crystals in DMSO properly. The 96-well plate reader (Flax station) measured the color intensity at 590 nm. Mean optical density of three replicate readings were plotted by normalizing each condition against an untreated control.

### Flow cytometric analysis

To measure mitochondrial superoxide, cells were stained with MitoSox Red dye (Molecular Probes: Cat# M36008) in culture media without Phenol Red at a final concentration of 5 μM for 30 min at 37 °C. Cells were washed two times in Ca^+2^/Mg^+2^ free PBS (Gibco: Cat#10,010–23) and resuspended in media without Phenol Red. For cytoplasmic superoxide staining, cells were stained with CellRox Deep Red dye (Molecular Probes: cat#C10422) in culture media without Phenol Red at a final concentration of 5 μM for 30 min at 37 °C. Cells were washed two times in Ca^+2^/Mg^+2^ free PBS, resuspended in media without Phenol Red. For intracellular ROS staining, cells were stained with CM-H2DCFDA dye (Thermo Fisher: cat#C6827) in culture media without Phenol Red at a final concentration of 5 μM for 30 min at 37 °C. Cells were washed two times in Ca^+2^/Mg^+2^ free PBS, and resuspended in media without Phenol Red. For lipid peroxide staining, cells were stained with Liperfluo dye (Dojindo: cat#50–190–3721) in culture media without Phenol Red at a final concentration of 5 μM for 30 min at 37 °C. Cells were washed two times in Ca^+2^/Mg^+2^ free PBS, and resuspended in media without Phenol Red. For mitochondrial labile iron staining, cells were stained with MitoFerro Green dye (Dijindo: Cat#M489) in culture media without Phenol Red at a final concentration of 5 μM for 30 min at 37 °C. Cells were washed two times in Ca^+2^/Mg^+2^ free PBS. For endoplasmic reticulum labile iron staining, cells were stained with FerroFar Red dye (Millipore: cat#SCT037) in culture media without Phenol Red at a final concentration of 5 μM for 30 min at 37 °C. Cells were washed two times in Ca^+2^/Mg^+2^ free PBS. For Golgi labile iron staining, cells were stained with BioTracker Red dye (Millipore: Cat#SCT030) in culture media without Phenol Red at a final concentration of 5 μM for 30 min at 37 °C. Cells were washed two times in Ca^+2^/Mg^+2^ free PBS. For cytoplasmic calcium staining, cells were stained with X-rhod1 AM dye (Molecular Probes: cat#14,210) in Ca^+2^/Mg^+2^ free PBS at a final concentration of 1 μM and 1 mM Probenecid (Molecular Probes, Cat#P36400) for 30 min at 37 °C. Cells were washed two times in Ca^+2^/Mg^+2^ free PBS, resuspended in PBS, and incubated further at 37 °C for 30 min to allow de-esterification of intracellular ester. For Mitochondrial calcium staining, cells were stained with Fluo4 AM dye (Molecular Probes: cat#14,201) in Ca^+2^/Mg^+2^ free PBS at a final concentration of 1 μM and 1 mM Probenecid (Molecular Probes, Cat#P36400) for 30 min at 37 °C. Cells were washed two times in Ca^+2^/Mg^+2^ free PBS, resuspended in PBS and incubated further at 37 °C for 30 min to allow de-esterification of intracellular ester. Once again cells were washed before acquiring. For apoptosis detection. equle concentration of Annexin V/7-AAD dyes was added in 100 μL binding buffer. The cells were incubated for 15 min at room temperature in the dark followed by addition of 400 μL binding buffer. The data were analyzed by flow cytometry within 1 hour. Mitochondrila membrane potential (ΔΨm) was studied using MitoView^TM^ dye in phenol red free media. The cells were incubated for 30 min at 37 °C followed by washing with Ca^2+^/Mg^2+^ free PBS. The cells were again resuspended in phenol red free media. The acquiring was done with a Fortessa flow cytometer (BD Bioscience) and data was analyzed using FACSDiva software (BD Bioscience). Mean fluorescence intensity was normalized with untreated parent cells.

### Proteomics and phosphoproteomic analysis

The experimental protocol followed was essentially as previously described and is outlined in supplementary Figure 1 [10]. Briefly, the samples were subjected to protein extraction followed by trypsin digestion. Tryptic peptides were labelled with TMT 6-plex reagents, and the pooled peptides were fractionated into 12 fractions using basic pH reversed-phase fractionation. Five percent of each fraction was used for global proteomics analysis while the remainder was subjected to phosphopeptide enrichment using Fe(III)‑NTA cartridges on an Agilent Bravo automated liquid handling platform. Fractions for global proteomics analysis and phosphoproteomic analysis were analyzed on an Orbitrap Exploris 480 mass spectrometer and UltiMate 3000 RSLC nano system (Thermo Scientific, San Jose, CA, USA). Fractions were resuspended in 0.1 % formic acid and injected on to a trap column (Optimize Technologies) using solvent A (0.1 % formic acid). Peptides were then separated on an analytical column (PepSep 40 cm × 100 µm, C_18_ 1.5 µm) for a total run time of 180 min using a gradient of solvent B (Acetonitrile, 0.1 % formic acid) from 5–35 % at 0.35 µL/min flow rate for 160 min. This is followed by increasing the solvent B to 90 % maintained for 9 min to wash the column. Mass spectrometry analysis was performed in data dependent mode in which a survey mass spectrometry scan was performed in an Orbitrap mass analyzer operated at 120,000 resolution. Precursor ions were collected with a normalized AGC target of 200 % or a maximum injection time of 50 ms and analyzed across a mass range of 350–1500 m*/z*. Precursor ions from charge states 2–6 were sequentially isolated based on abundance using quadrupole mass filter with an isolation width of 0.7 m*/z*. Isolated precursor ions were fragmented using HCD mode with a normalized collision energy of 34 %. Fragmented ions were collected for a normalized AGC target of 200 % or a maximum injection time of 100 ms and recorded in an Orbitrap mass analyzer at 30,000 resolution. Dynamic exclusion setting was enabled with an exclusion duration of 35 s and exclusion mass width of 10 ppm.

For the analysis of phosphopeptide fractions, a gradient of 5–35 % solvent B was used for separating the phosphopeptides for 135 min followed by a high organic wash of 90 % solvent B for 5 min. An overall run time of 150 min was used for the analysis. Mass spectrometry analysis was performed as described above except an isolation width of 1.2 m*/z* was used for isolating the precursor ions for MS/MS analysis.

Mass spectrometry raw data from proteomic and phosphoproteomic analyses were analyzed using the Sequest HT search engine in Proteome Discoverer software (version 2.5). MS/MS spectra was searched against the human Uniprot protein database with full tryptic cleavage specificity, two missed cleavages, precursor mass tolerance of 10 ppm and fragment mass tolerance of 0.05 Da. Oxidation (M), acetylation (protein N-terminus) as dynamic modification and carbamidomethylation (C), TMT modification (K, peptide N-terminus) were specified. Additionally, phosphorylation (S,T,Y) was specified as a dynamic modification for the phosphoproteomics data. Percolator was used for the false discovery rate calculation which was maintained at 1 % for protein and peptide levels. Reporter ion quantitation was performed by integrating the signal with 20 ppm tolerance. PSMs with an average report ion S/N threshold of 10 were filtered out for quantitation. Finally, normalized abundances were calculated by using total peptide amount option which were used for further data analysis.

In summary, protein samples were digested, TMT-labeled, and fractionated for total proteomics and phosphoproteomic analyses. Enrichment and analysis of phosphopeptides were done using the Agilent system and Orbitrap Exploris 480, respectively. The identified phosphopeptides with normalized abundance values reflected the relative expression across samples.

## Data analysis

The data were analyzed in Prism 8 (GraphPad Software, San Diego, CA, USA). Ingenuity pathway analysis (IPA) was performed on differentially expressed proteins comparing untreated versus treated groups. The networks, functional analyses and graphical summary were generated using QIAGEN IPA (QIAGEN Inc., https://digitalinsights.qiagen.com/IPA). The default parameters were used for data interpretation. The phosphoproteomics data was analyzed using a “Phosphomatics” webtool available online (https://phosphomatics.com). Kinase-Substrate Enrichment Analysis (KSEA) was performed as described by Casado et.al [11].

## Results

### Iron chelators are most potent killers of multiple myeloma cell lines

**Figure 1:**
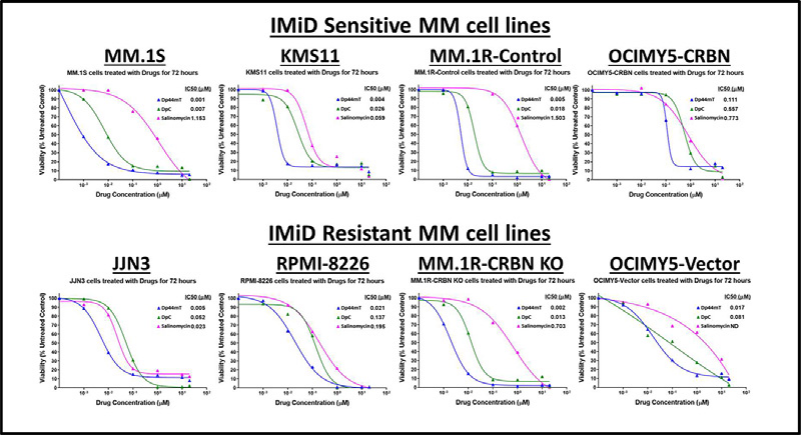
Iron chelators are potent killers of multiple myeloid cell lines. Multiple myeloid (MM) cell-lines (IMiD sensitive and IMiD resistant) were treated with Drugs (Dp44mT, DpC and Salinomycin) for 72 h. MTT assay was performed at the end of 72 h incubation and half maximal inhibitory concentration (IC_50_) values were determined using GraphPad Prism software.

The antiproliferative activity of various drugs was investigated in MM cells. Of the tested compounds, the most potent drugs found to kill MM cell lines, came under one common umbrella of iron chelators (data not shown). Therefore, three different iron chelators were tested, namely: Dp44mT, DpC, and Salinomycin. All of these drugs were effective in killing MM cells in exceptionally low concentrations. Interestingly, the effect of iron chelators was stronger than the clinically approved immunomodulatory drugs, and their effect was independent of *CRBN* expression (experiments with cereblon-knockout cells; data not shown). Of the iron chelators, Dp44mT exhibited the most potent ability to kill MM cell lines with highest potency (half maximal inhibitory concentration [IC_50_] = 0.001 to −0.1 μM - Figure 1).

In comparison, treatment with IMiDs did not induce 100 % killing in MM cell lines. Only IMiD sensitive cell lines - *MM.1S, KMS11, MM.1R, OCIMY5-CRBN*-overexpressed showed sensitivity to lenalidomide with saturation at certain concentrations, and once the drug reached its maximum anti-proliferation effect, there was no further killing even by increasing the lenalidomide concentration to as high as 100 μM. As anticipated, IMiD resistant cell lines *JJN3, RPMI-8226, MM.1R CRBN-KO* showed no response to IMiD treatment (Data not shown).

These data suggest a potent anti-MM effect of Dp44mT and similar redox-active iron chelators that can be effective even in IMiD -resistant clones. (Figure 1).

### Dp44mT-induced proteomic changes in multiple myeloma cell lines

To firmly establish the mechanism of the anti-MM effect of Dp44mT, global proteomics- and phosphoproteomics-based evaluations of molecular pathways were adopted. Two MM cell lines, *MM.1S* (IMiD sensitive), and *RPMI-8226* (IMiD resistant) were selected and treated with Dp44mT for two different time periods (24 and 48-hours). Two concentrations were selected from cell viability data obtained from studies with Dp44mT treatment for 72 h; the concentration at which ∼90 % cells died at 72 h i.e., 0.1 nM and the concentration at which ∼50 % cells died after 72 h of Dp44mT treatment i.e., 0.01 nM. The cells were treated with or without Dp44mT and pellets were collected first at 24 h and then after 48 h for downstream experiments.

The global proteomic analysis identified 8066 proteins in the *MM.1S* cell line and 7972 proteins in the *RPMI-8226* cell line. Principal component analysis for both cell lines revealed a substantial variability after Dp44mT treatment. The normalized abundance ratios were used to calculate the fold change between the groups with the respective untreated group. The most prominent proteomics expression level changes were observed at the 48-hour time point at both studied concentrations i.e., 0.1 µM and 0.01 µM.

**Figure 2:**
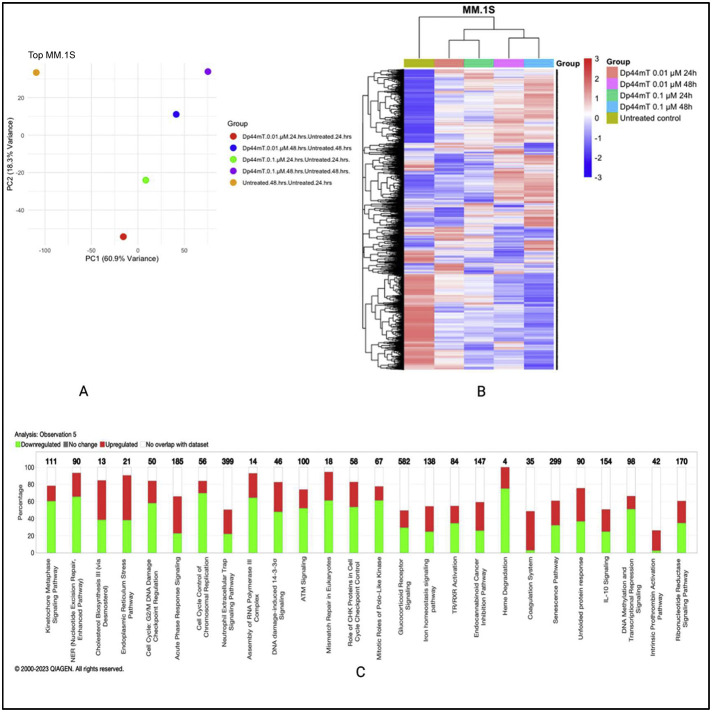

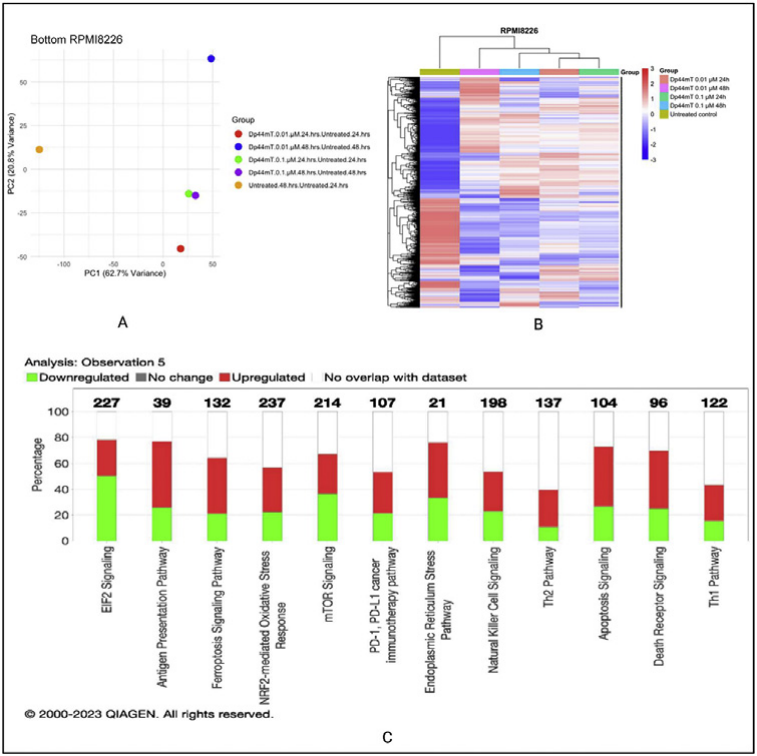
Dp44mT-induced proteomic changes in multiple myeloid (MM) cell lines: A. Principal component analysis. Unit variance scaling was employed to rows and the principal components were calculated using the singular value decomposition imputation approach. The X and Y axes correspond to principal component (PC)-1 and PC-2, respectively thus explaining the total variance (*n* = 5) data points. B. Heatmap of differentially regulated proteins after Dp44mT treatment (Only those genes with absolute fold change |log2(FC)| >1.5 were included to reduce the size of the data). C. Canonical ingenuity pathway analysis showing differentially regulated pathways in MM cell lines. Green represents downregulation, red represents upregulation, and white represents no overlap with the ingenuity pathway knowledge-based database. *MM.1S* (top) and *RPMI-8226* (bottom).

Ingenuity pathway analysis highlighted differentially expressed pathways specific to the *MM.1S* cell line such as cell cycle, control of chromosomal replication, ATM signaling, and unfolded protein response, amongst others. On the other hand, the *RPMI-8226* cell line showed differential expression involving pathways such as ferroptosis signaling, glutathione biosynthesis, and death receptor signaling. Given that both cell lines included in this study for proteomics and phosphoproteomics analyses differ a lot in their underlying biology, the mechanism of action of Dp44mT also showed some differences between them (Figure 2). The graphical summary of biological processes and their activation (Orange) or inhibition (Blue) state in Dp44mt treated versus untreated cell lines are shown in Supplementary Figure 2 A & B. The similar/crosstalk biological processes represented in both cell lines were related to iron homeostasis and endoplasmic reticulum stress. The data of this study show evidence of the ability of Dp44mT to modulate iron metabolism by iron starvation, decrease antioxidant defenses via NRF2-mediated oxidative stress response, and increase endoplasmic reticulum stress in MM cell lines.

However, considering the lack of an obvious common biological pathway to explain the anti-MM effect of Dp44mT between the two cell lines, further studies with phosphoproteomics were undertaken.

### Global phosphoproteomics after Dp44mT treatment establishes a link with iron metabolism

Iron plays an important role in reprogramming phosphorylation signaling [12]. Phosphorylation is a post-translational modification that has a crucial role in regulating protein function and cellular signaling.

To investigate Dp44mT-induced phosphoproteomic alterations in IMiD resistant and IMiD sensitive cell lines, phosphopeptides identified from the *MM.1S* and *RPMI-8226* cell lines were measured using TMT-based multiplexed quantitation. Approximately ∼27,000 phosphopeptides were identified in both experiments. The phosphoproteins and phosphosites with fold changes |(FC)| >1.5 were identified as differentially expressed phosphosites. After normalization and filtering out missing data, ∼17,000 phosphorylation sites were found to be present in each group with approximately ∼5000 unique proteins (Supplementary Figure 3 A-F).

The top active kinases after 48 h of Dp44mT treatment of the *MM.1S* cell line include CDK1 AURKB1, AURKB, CDK2, and TTK. CSNK2A2, ATM, AKT1, PRKD1 and SGK1 were more active in the untreated groups. Most of the top kinases identified in the Dp44mT treatment group are involved in controlling cellular processes necessary for cell growth, survival, and DNA damage response.

On the contrary, the top downregulated kinase CSNK2A2 is the catalytic subunit of the protein kinase CK2 (formerly known as casein kinase 2). CK2 has been demonstrated to interact with and modify the activity of iron-related proteins, such as the iron regulatory protein 1 (IRP1) and the iron-sulfur cluster assembly enzyme ISCU [13]. Abnormal expression of *CSNK2A2* has been implicated in various diseases, including cancer [14]. Moreover, the downregulation of kinases like ATM and AKT1 might make cells more vulnerable to damage, leading to cell death.

The *RPMI-8226* cell line showed CDK2, PRKCD, AKT1, MAPK8, PRKD1, CDK4 as most represented kinases in the Dp44mT treatment group and SRPK1, HIPK2, CDK9, PRKG1, MAPK3, in the untreated group. These protein kinases contribute in various signaling pathways, and regulatory mechanisms. Their functions are diverse and can include roles in cell cycle regulation, transcriptional control, signal transduction, apoptosis, and cellular growth and survival.

**Figure 3:**
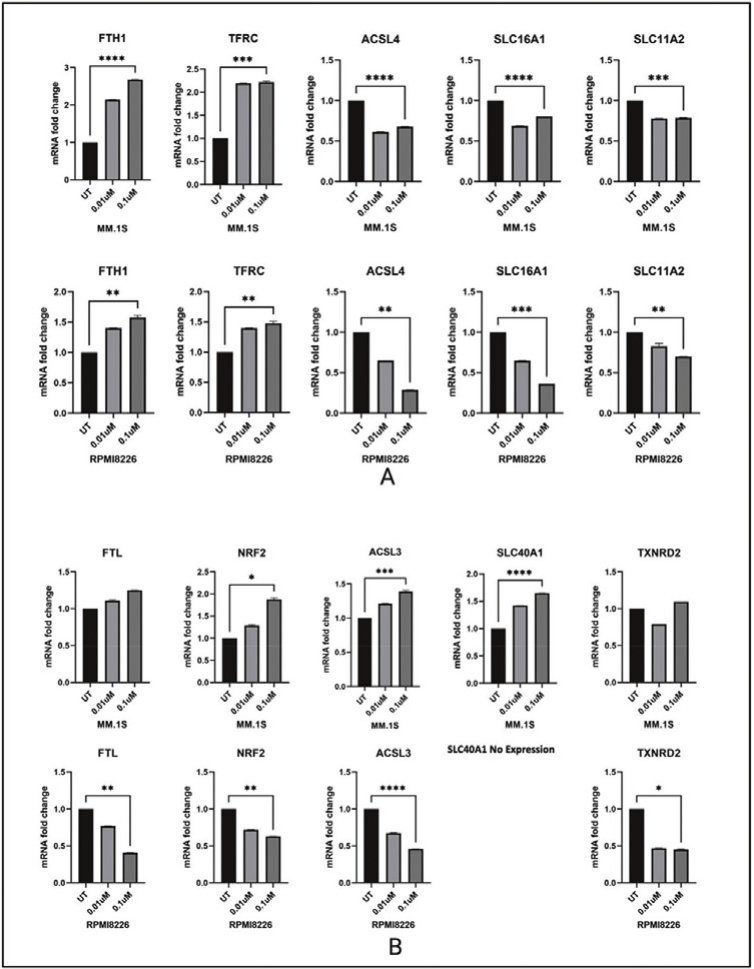
Global Phosphoproteomics changes after Dp44mT treatment: A & D: Kinase-substrate enrichment analysis (as described by Casado et al. and Wiredja et al.*)* the plot shows the Z-score for enrichment of different kinases in present data. Kinases with Z-scores greater than 0 are more active in the control group (Untreated group). B & E: Upstream motif analysis to identify kinases responsible for phosphorylation. C & F: Enriched pathways of most represented phosphorites. *MM.1S* (Top) and *RPMI-8226* (Bottom).

Of interest, the enrichment analysis showed over representation of spliceosomes, autophagy, AMPK pathway, cell cycle, and protein processing in endoplasmic reticulum of both cell lines. All the above pathways are critical aspects of cellular physiology. AMPK regulates cellular energy homeostasis. It functions as a sensor of AMP/ATP ratios, activating energy-generating processes and inhibiting energy-consuming pathways to restore energy balance. This is in line with a previous study that concluded that activation of the AMPK pathway after Dp44mT treatment is a cellular mechanism to rescue the loss of iron and to deal with oxidative stress generated by Dp44mT [15]. Additionally, dysregulation of fundamental cellular processes like cell cycle and protein processing in endoplasmic reticulum after Dp44mT treatment points towards the potential of novel mechanisms for anti-tumor activity of Dp44mT (Figure 3A-F).

### Dp44mT-induced changes in levels of major iron regulating genes

Since Dp44mT is known to target the iron metabolism, and both AMPK and endoplasmic reticulum stress are implicated in ferroptosis [16], Dp44mT-induced changes in the expression of major iron metabolism genes were determined. The mRNA of ten genes involved in major iron metabolism-related biological processes were quantified. Change in iron storage (*FTH1* and *FTL*), iron transport (*SLC11A2, SLC40A1* and *SLC16A1*), iron uptake (*TFRC*), oxidative stress defense (*NRF2*), redox regulation (*TXNRD2*), and ferroptosis (*ACSL3* and *ACSL4*) were measured.

The *MM.1S* (IMiD sensitive) and *RPMI-8226* (IMiD resistant) cell lines showed distinct gene expression profiles of iron metabolism genes after 24 and 48 h of Dp44mT treatment. The common differential expression pattern observed between both cell lines was seen in genes like *FTH1, TFRC, ACSL4, SLC11A2, SLC16A1. NRF2, SLC40A1* (*RPMI-8226* lack *SLC40A1* gene expression), *ACSL3, FTL* showed opposite trends in expression after Dp44mT treatment. The observed cell death after Dp44mT treatment could primarily be due to iron mis-localization and deprivation in some cellular compartments. Both iron depletion and iron excess can trigger apoptotic cell death through disrupted redox balance, mitochondrial dysfunction, and activation of apoptotic pathways. The ability of Dp44mT to chelate iron can lead to cellular iron maldistribution ultimately leading to cell death.

It is possible that a constitutive lack of expression of the iron export protein SLC40A1 (ferroportin) in the *RPMI-8226* cell line could lead to compensatory mechanisms to deal with iron dysregulation making Dp44mT less effective compared to its effect on other MM cell lines. The IC_50_ of the *RPMI-8226* cell line was 0.02 compared to 0.001 for the *MM.1S* cell line. A decrease in ferroportin expression has been reported to promote myeloma growth in previous research articles [17,18].

**Figure 4:**
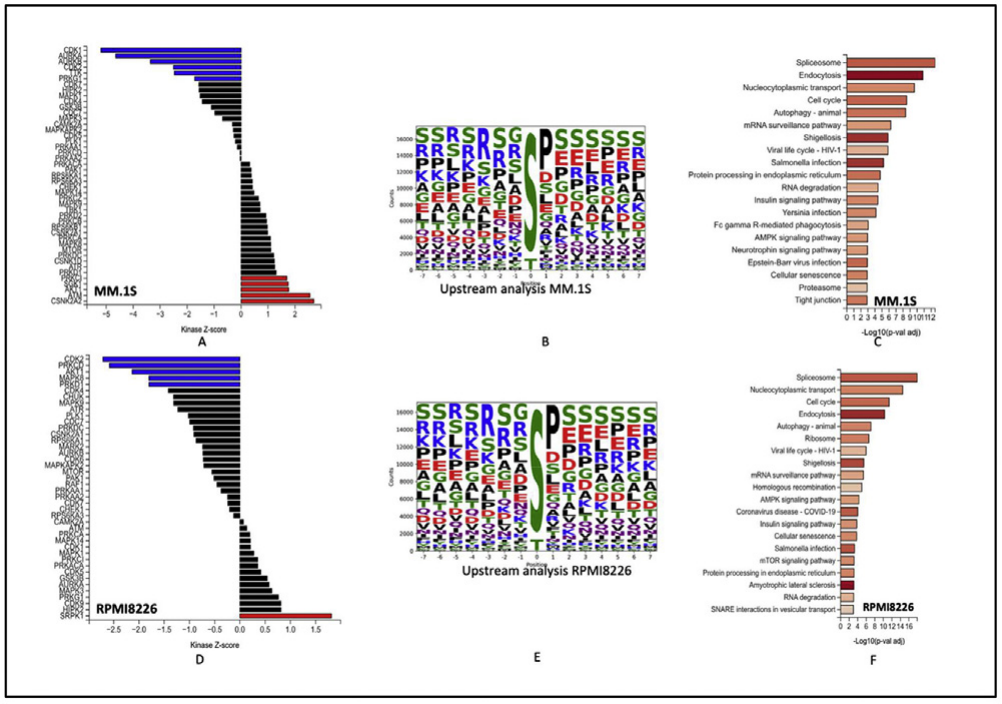
Changes in major iron metabolism related genes 48 h after Dp44mT treatment in *MM.1S* and *RPMI-8226* cell lines. A: The genes with similar expression patterns in both studied cell lines are shown at the top and B: Genes having differential expression patterns in response to Dp44mT treatment are shown at the bottom.

Of interest, there was substantial downregulation of the ferroptosis mediating gene *ACSL4* in both cell lines after Dp44mT treatment. *ACSL4* is a highly expressed gene in MM and its downregulation in response to Dp44mT might suggest a dual function. A recent study by Zhang et al. has also experimentally proven that *ACSL4* is abnormally expressed in MM patients and directly promotes MM cell proliferation. Further, knockdown of *ACSL4* suppressed MM cell viability despite reducing susceptibility to RSL3-induced ferroptosis [19]. Thus, suppression of *ACSL4* may be a key mechanism through which Dp44mT mediates its anti-myeloma effect. Collectively, data suggest that the disordered iron metabolism in MM cell lines can be a therapeutic target in the development of effective therapies against MM progression (Figure 4).

### Dp44mT-induced multiple myeloma cell apoptosis

**Figure 5:**
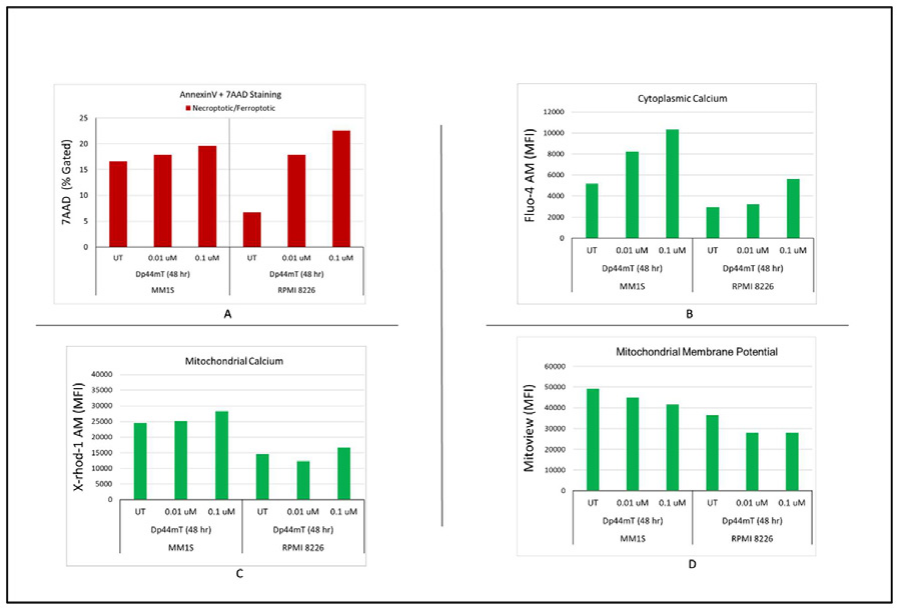
Dp44mT-induced multiple myeloid (MM) cell apoptosis: Flow cytometric analyses to detect the apoptotic profiles of MM cells. A: Annexin 5/7AAD was used to quantify the apoptotic cells. B & C: The increased influx of cytosolic calcium and decrease in mitochondrial calcium is likely due to activation of apoptosis under Dp44mT-induced stress D: Time- and Dose-dependent decreases in mitochondrial membrane potential (ΔΨm) after Dp44mT treatment.

A flow cytometric approach was utilized to explore the underling mechanism of the Dp44mT-induced anti-proliferative effect to find the apoptotic profiles of MM cells. Annexin 5/7AAD was used to quantify apoptotic cells. The data showed that Dp44mT (0.1 and 0.01 µM) significantly increased the number of apoptotic cells in both tested cell lines following a distinct dose-dependent manner. Moreover, a decrease in mitochondrial calcium and an increase in cytoplasmic calcium were also observed in both cell lines at both time points and concentrations. The increased influx of cytosolic calcium and decrease in mitochondrial calcium is likely due to activation of apoptosis under Dp44mT-induced stress (Figure 5A–C).

### Time- and dose-dependent mitochondrial membrane potential (ΔΨm) after Dp44mT treatment

To study the effect of Dp44mT treatment on overall mitochondrial health, the ΔΨm was measured using Mitoview dye. A decrease in ΔΨm was observed in both cell lines. A decrease in ΔΨm after Dp44mT treatment indicates the activation of early events in the intrinsic pathway of apoptosis. These results indicate that Dp44mT impairs mitochondrial function, thereby weakening the capacity to maintain the proton gradient across the inner mitochondrial membrane, a process that ultimately leads to cell death (Figure 5D).

### Dp44mT-induced imbalance in reactive oxygen species homeostasis

**Figure 6:**
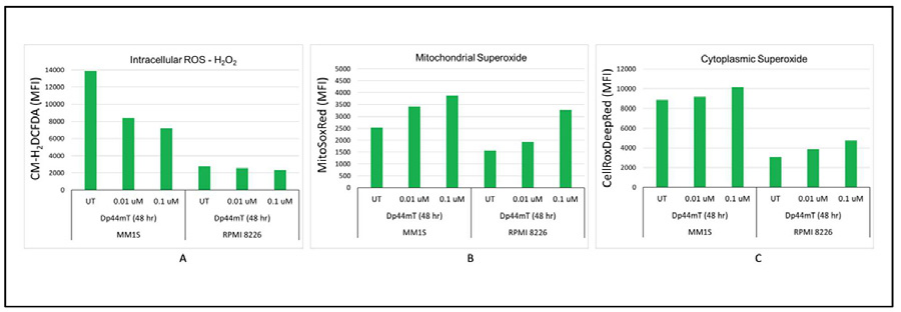
Dp44mT-induced imbalanced reactive oxygen species (ROS) homeostasis: A: Time- and dose-dependent decreases in intracellular ROS levels after Dp44mT treatment of the IMiD sensitive cell line (*MM.1S*). However, the IMiD resistant cell line (*RPMI-8226*) did not show any change in intracellular ROS levels after Dp44mT treatment. B & C: Time- and dose-dependent increases in mitochondrial and cytoplasmic superoxide in both cell lines.

Dp44mT lies among thiosemicarbazones that are actively involved in redox cycling of their bound iron and the generation of ROS. Previous studies have proved that Dp44mT can induce ROS accumulation, leading to increased cytotoxicity [15,20]. To test this, intracellular H_2_O_2_ was measured by flow cytometry using the H2DCF-DA dye, which becomes fluorescent when oxidized by O_2_^-^, H_2_O_2_, or HO^-^. Interestingly, time- and dose-dependent decreases in intracellular ROS levels were observed after Dp44mT treatment in the IMiD sensitive cell line (*MM.1S*). However, the IMiD resistant cell line (*RPMI-8226*) did not show any change in intracellular ROS levels after Dp44mT treatment. On the other hand, time- and dose-dependent increases in mitochondrial and cytoplasmic superoxide were observed. The combination of increased superoxide and decreased H_2_O_2_ suggests a shift in the balance of ROS metabolism that is cellular compartment-specific (Figure 6A-C).

Lipid peroxidation levels after DP44mT treatment in MM cells were measured to get deeper insights into the mechanisms underlying oxidative damage and the disease processes. There were increases in lipid peroxidation levels with the IMiD sensitive cell line (*MM.1S*) at both time points and concentrations. However, the results for the IMiD resistant cell line (*RPMI-8226*) cells were inconsistent and did not show any specific pattern (Supplementary Figure 4A). This observed pattern suggests a complex oxidative stress response where increased superoxide levels are leading to lipid peroxidation in the IMiD sensitive cell line. Increased lipid peroxidation despite a reduction in *ACSL4* expression indicates alternate pathways for this effect as shown before [21]. Thus, Dp44mT might mediate MM cell death both through ferroptosis and via an independent effect mediated by *ACSL4* suppression.

Furthermore, labile iron compartmentalization was quantified by measuring the labile iron levels within the Golgi complex and the endoplasmic reticulum. Some dysregulation in labile iron levels was observed, but the results did not show any specific pattern (Supplementary Figure 4B-C).

## Discussion

Iron chelators have obtained considerable attention as potential anti-cancer agents in few decades due to their ability to target iron-dependent processes that are essential for cancer cell growth. Cancer cells have a higher requirement for iron than healthy cells because they proliferate rapidly. In the present study, the potential of redox-active iron chelator Dp44mT in killing MM cell lines was confirmed. The anti-myeloma potential of Dp44mT was found to be independent of *CRBN* expression or IMiD sensitivity. The drug was found to be effective in nanomolar concentrations. Specific proteins/phosphosites that are altered in response to DP44mT treatment were identified by comparing the total proteome and phosphoproteome of DP44mT-treated cells with untreated control cells. The disruption of cellular iron metabolism, ROS homeostasis, and ΔΨm was also substantially characterized as part of the mechanism of action for the Dp44mT-induced anti-myeloma effect. This information may help to elucidate the molecular mechanisms underlying the effects of the compound and potentially identify key signaling pathways affected by DP44mT.

Phosphoproteomics data showed significant enrichment of the AMPK pathway, cell cycle, protein processing in the endoplasmic reticulum in both cell lines after D44mT treatment. All the above pathways are critical aspects of cellular physiology including ferroptosis [22,23]. These pathways are interconnected, and their dysregulation can have significant implications for cellular function and disease processes. For example, AMPK can influence the cell cycle by regulating key cell cycle proteins and checkpoint control and can also regulate ferroptosis [22,24]. Dysregulation of protein processing in the endoplasmic reticulum can also impact cellular proteostasis and contribute to cell death including ferroptosis [25,26]. Understanding the molecular mechanisms underlying these pathways and their interplay is crucial for developing targeted interventions and therapies to treat various diseases, including cancer, metabolic disorders, and protein misfolding diseases.

An imbalance of the ROS metabolism, decrease in ΔΨm, and disruption in calcium efflux were also observed. These results demonstrate that Dp44mT disturbs intracellular redox balance thereby activating apoptosis. Malignant transformation of MM has already been linked with overproduction of intracellular ROS because of enhanced metabolism in tumor cells [27]. However, cells adapt to deal with oxidative stress by activating alternate mechanisms. Hence, further induction of oxidative stress can be an effective strategy to manage MM. A previous study proved that MM cell lines with decreased antioxidant defense are more vulnerable to IMiD-induced oxidative stress therefore highlighting the need of further exploring redox balance in MM cells [28]. Many studies have confirmed that Dp44mT, different from other iron chelators like deferoxamine, can generate ROS by a process called redox cycling: it therefore falls under the category of a redox-active compound [29]. Taken together Dp44mT, exhibits a dual role in killing MM cells by starving MM cells from iron and by the overproduction of ROS. An increase in lipid peroxidation was also observed despite a reduction in *ACSL4* expression, thus indicating alternate *ACSL4*-independent pathways for lipid peroxidation as shown previously. Thus, Dp44mT could mediate MM cell death both through ferroptosis and via an independent effect mediated by *ACSL4* suppression.

DP44mT has been the subject of numerous studies investigating its potential as an anti-cancer drug. A study by Lovejoy et al. in 2002 described the initial characterization of DP44mT and its analogs as novel iron chelators with potent antiproliferative activity against cancer cells. In the study, the researchers investigated the anti-cancer properties of DP44mT and its analogs as novel iron chelators. They aimed to explore the potential of these compounds to disrupt iron metabolism and selectively inhibit cancer cell growth [30]. Since then, there have been numerous research articles published related to the potential of Dp44mT as a potent anti-cancer drug. Another study by Whitnall et al. showed that DP44mT and its analogs overcome resistance to standard chemotherapeutic agents in cancer cells. The findings suggest that DP44mT and its derivatives have broad-spectrum antitumor activity and the potential to enhance the effectiveness of conventional chemotherapeutic drugs [31]. To study the underlying molecular mechanism behind Dp44mT, Dixon et al. investigated the molecular mechanisms underlying the anti-cancer effects of DP44mT in prostate cancer cells. The researchers demonstrated that DP44mT targets multiple signaling pathways, including AKT, TGF-β, and ERK, and engages the metastasis suppressor *NDRG1*, leading to inhibition of cell growth and metastasis [32].

These references provide a glimpse into the research on DP44mT as an anti-cancer drug. However, it is important to note that the scientific literature that has investigated the properties, mechanisms of action, and therapeutic potential of DP44mT in different cancer models and contexts has reported divergent findings. Therefore, it must be understood that Dp44mT can influence multiple pathways or biological processes which are highly dependent on the underlying biology of the study subject. However, the findings of this study provide novel insights, establishing a potential link between previously disparate findings via the dysregulation of iron metabolism, lipid peroxidation, and the inhibition of *ACSL4* expression. Iron must be tightly regulated for any cell to function properly and any disruption of iron machinery, whether it is upregulation or downregulation, can be detrimental. Therefore, targeting iron in MM is an excellent approach to develop anti-myeloma therapies.

## Conclusion

In-vitro anti-MM effect of Dp44mT appears to be mediated by dysregulated iron metabolism, ROS, and other biological pathways. The present study highlights the antitumor role of Dp44mT and its likely therapeutic potential that could be tested in future clinical trial studies.

## Conflicts of interest

All authors declare no conflict of Interest.

## Appendix Supplementary materials

Image, application 1
