# Supplementary figures and images for: Mechanistic insights into the antiproliferative effect of the redox-active iron chelator Dp44mT on multiple myeloma cell lines
Source: Hematol Transfus Cell Ther. 2025 Dec 23;48(1):106233. doi: 10.1016/j.htct.2025.106233 (PMC12800591; doi:10.1016/j.htct.2025.106233)

**Supplementary Figure 1:**

**
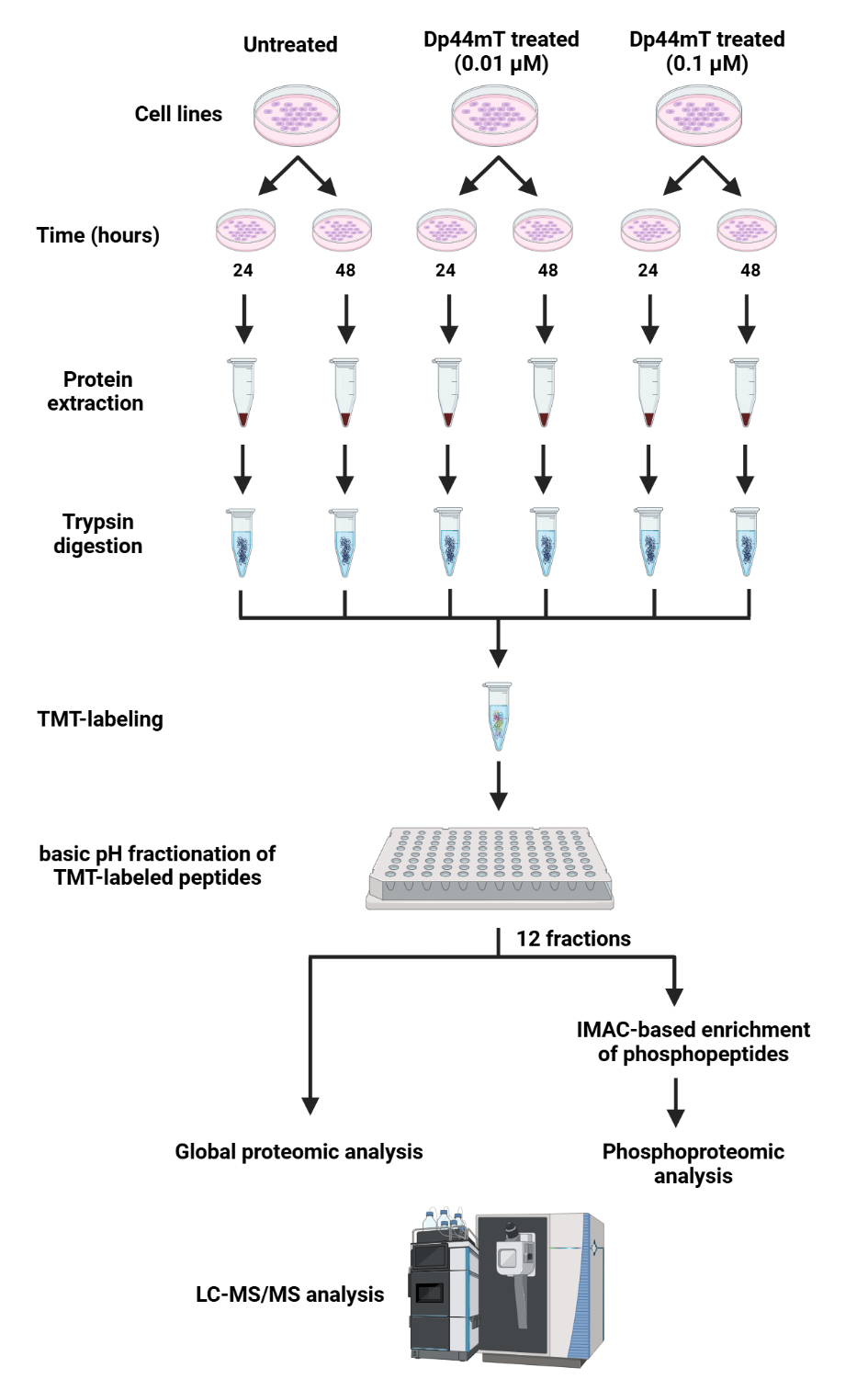
**

**Supplementary Figure 2:**


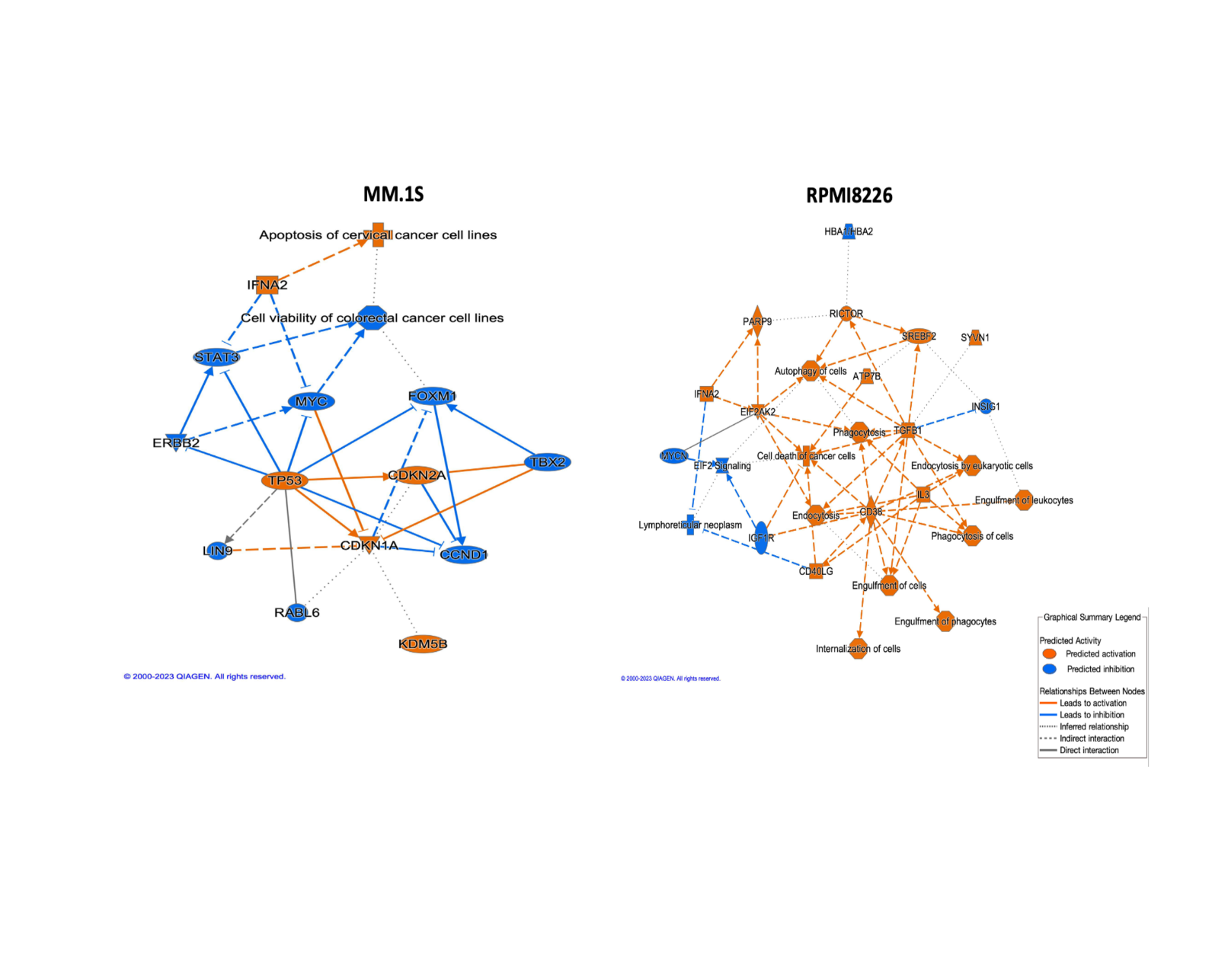


**Supplementary Figure 3:**


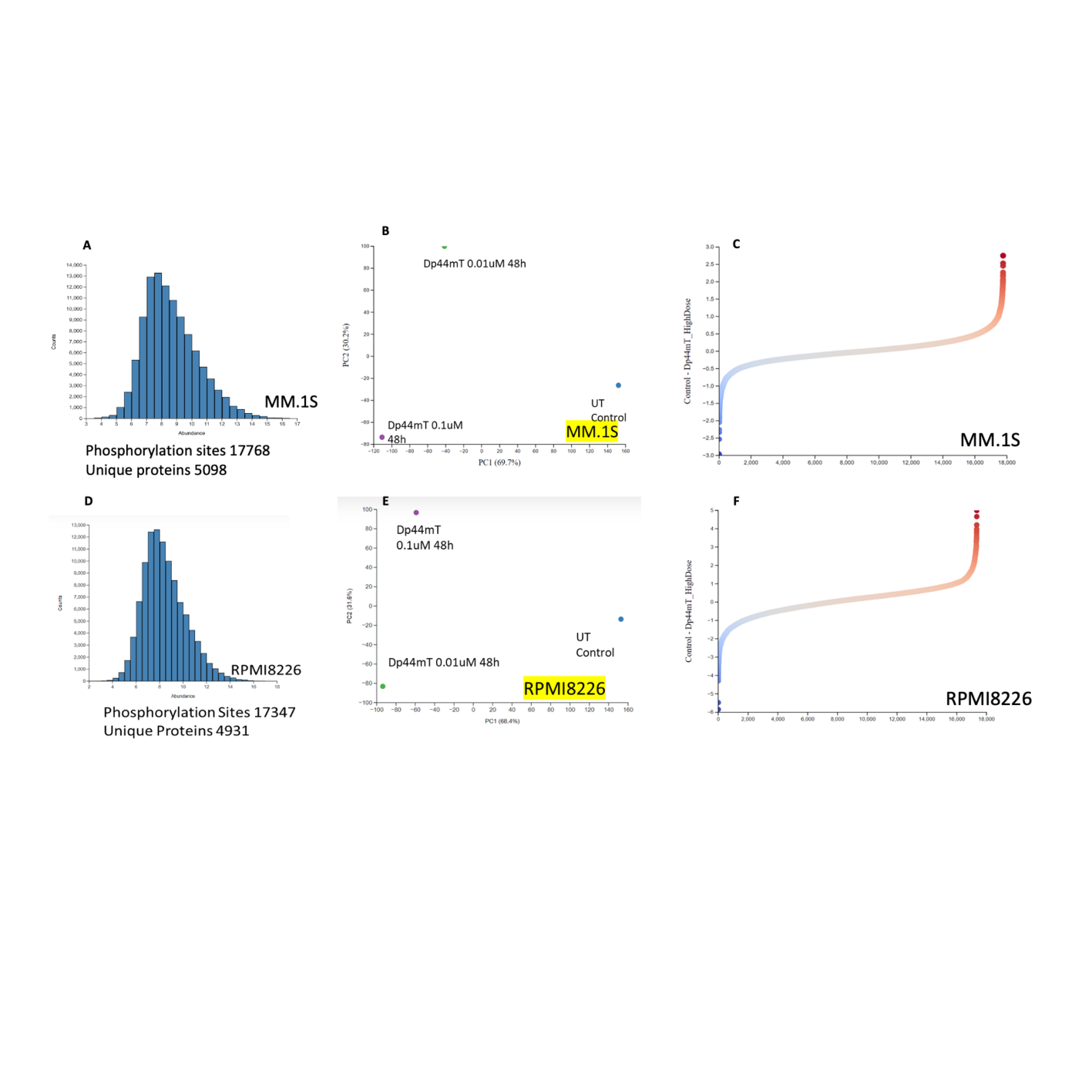


**Supplementary Figure 4:**


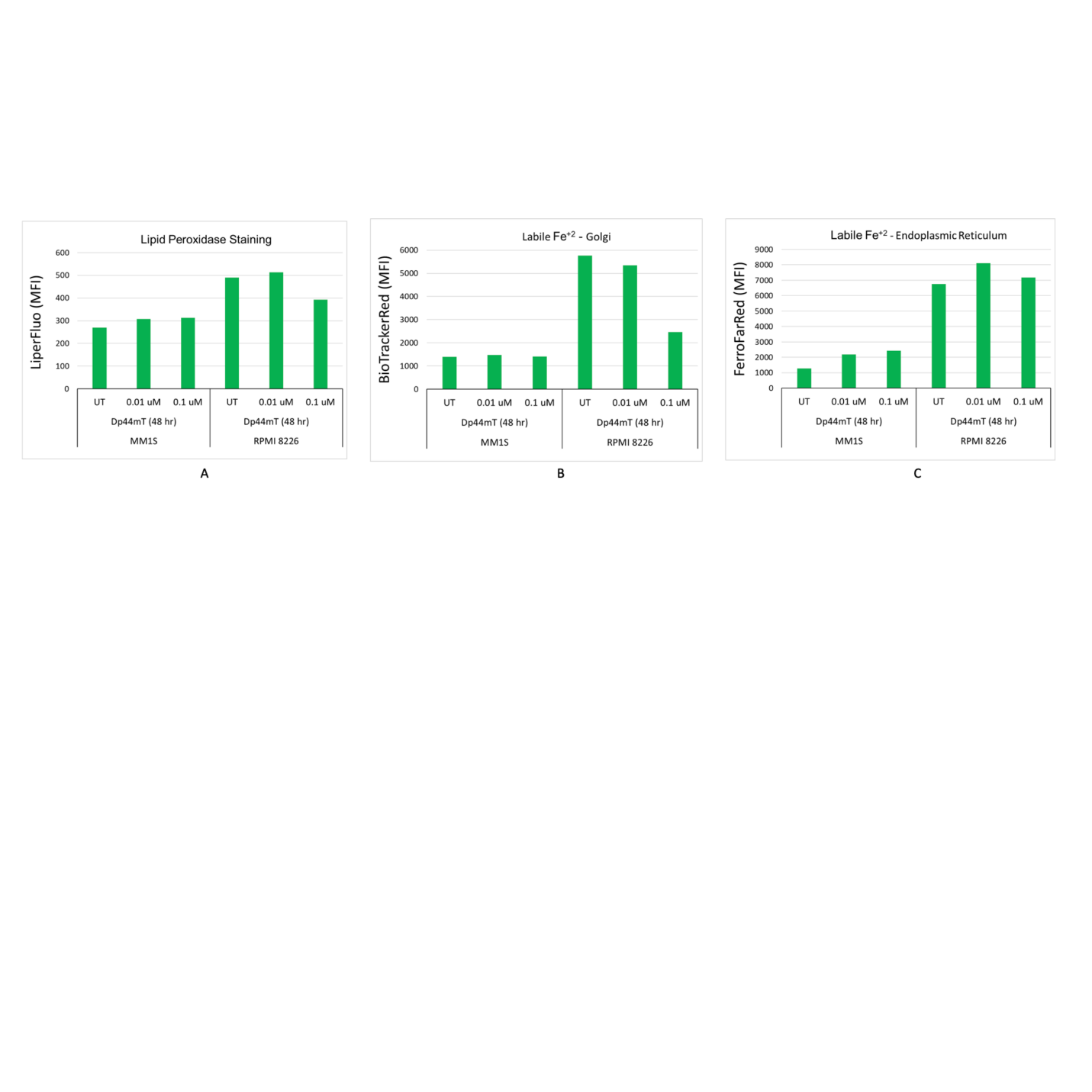

Supplement: Supplementary file 1 [file mmc1.docx]
